# Evidence-based clinical practice guidelines for nonalcoholic fatty liver disease/nonalcoholic steatohepatitis 2020

**DOI:** 10.1007/s00535-021-01796-x

**Published:** 2021-09-17

**Authors:** Katsutoshi Tokushige, Kenichi Ikejima, Masafumi Ono, Yuichiro Eguchi, Yoshihiro Kamada, Yoshito Itoh, Norio Akuta, Masato Yoneda, Motoh Iwasa, Masashi Yoneda, Motoyuki Otsuka, Nobuharu Tamaki, Tomomi Kogiso, Hiroto Miwa, Kazuaki Chayama, Nobuyuki Enomoto, Tooru Shimosegawa, Tetsuo Takehara, Kazuhiko Koike

**Affiliations:** 1 Guidelines Committee for Creating and Evaluating the ‘‘Evidence-Based Clinical Practice Guidelines for Nonalcoholic Fatty Liver Disease/Nonalcoholic Steatohepatitis’’, The Japanese Society of Gastroenterology / The Japan Society of Hepatology, 6F Shimbashi i-MARK Building, 2-6-2 Shimbashi, Minato-ku, Tokyo, 105-0004 Japan; 2 The Japan Society of Hepatology, Kashiwaya 2 Building 5F, 3-28-10 Hongo, Bunkyo-ku, Tokyo, 113-0033 Japan; 3 Institute of Gastroenterology, Department of Internal Medicine, Tokyo Women’s Medical University, 8-1 Kawada-cho, Shinjuku-ku, Tokyo, Japan

**Keywords:** NAFLD/NASH guidelines, Fibrosis, HCC, Cardiovascular disease, Treatment

## Abstract

Nonalcoholic fatty liver disease (NAFLD) has become a serious public health issue not only in Western countries but also in Japan. Within the wide spectrum of NAFLD, nonalcoholic steatohepatitis (NASH) is a progressive form of disease that often develops into liver cirrhosis and increases the risk of hepatocellular carcinoma (HCC). While a definite diagnosis of NASH requires liver biopsy to confirm the presence of hepatocyte ballooning, hepatic fibrosis is the most important prognostic factor in NAFLD. With so many NAFLD patients, it is essential to have an effective screening method for NAFLD with hepatic fibrosis. As HCC with non-viral liver disease has increased markedly in Japan, effective screening and surveillance of HCC are also urgently needed. The most common death etiology in NAFLD patients is cardiovascular disease (CVD) event. Gastroenterologists must, therefore, pay close attention to CVD when examining NAFLD patients. In the updated guidelines, we propose screening and follow-up methods for hepatic fibrosis, HCC, and CVD in NAFLD patients. Several drug trials are ongoing for NAFLD/NASH therapy, however, there is currently no specific drug therapy for NAFLD/NASH. In addition to vitamin E and thiazolidinedione derivatives, recent trials have focused on sodium glucose co-transporter 2 (SGLT2) inhibitors and glucagon-like peptide-1 (GLP-1) analogues, and effective therapies are expected to be developed. These practical guidelines for NAFLD/NASH were established by the Japanese Society of Gastroenterology in conjunction with the Japan Society of Hepatology. Clinical evidence reported internationally between 1983 and October 2018 was collected, and each clinical and background question was evaluated using the Grades of Recommendation Assessment, Development and Evaluation (GRADE) system. This English summary provides the core essentials of these clinical practice guidelines, which include the definition and concept, screening systems for hepatic fibrosis, HCC and CVD, and current therapies for NAFLD/NASH in Japan.

## Introduction

Nonalcoholic fatty liver disease (NAFLD) has become the most prevalent cause of chronic liver disease worldwide [1] and is now the fastest-growing indication for liver transplantation among waitlist registrants [2]. There are more than 20 million NAFLD patients in Japan, and it is feared that this number will increase in the future [3]. In recent years, hepatocellular carcinoma (HCC) based on non-viral liver disease has increased, and the need for a screening method has become urgent.

The Japanese NAFLD/NASH guidelines were established in 2014 [4]. These clinical guidelines have received considerable attention and been widely used. However, new knowledge has since been reported relating to their concepts, diagnostic imaging methods, and treatment methods. Therefore, a joint committee of the Japanese Society of Gastroenterology and the Japanese Society of Hepatology has reviewed and revised these guidelines.

The current guidelines summarize reports from 1983 to the end of October 2018, focusing on the following: (1) concept and clinical significance of liver fibrosis, (2) screening and follow-up systems for liver fibrosis, (3) surveillance of HCC, (4) a system for consultation with specialists regarding cardiovascular risk in NAFLD, and (5) new therapeutics. We hope that these guidelines will be used widely in clinical practice, and also hope to discuss and improve any problems that may arise in the clinical field so that further revisions may be made as necessary.

## The concept and definition of NAFLD

In the 2014 guidelines [4], NAFLD was characterized by evidence of hepatic steatosis by either imaging or histology and the appropriate exclusion of other liver diseases. NAFLD is histologically characterized by macrovesicular steatosis and further categorized into nonalcoholic fatty liver (NAFL) and nonalcoholic steatohepatitis (NASH). In the revised guidelines, the concept and diagnosis of NAFLD are essentially the same (Table 1).

**Table 1:** New definition and concept of NAFLD

Nonalcoholic fatty liver disease (NAFLD) is characterized by evidence of hepatic steatosis as determined by either imaging or histology, associated with metabolic factors. Other liver diseases, such as alcoholic liver disease, viral liver disease, and drug-induced liver disorder are excluded. NAFLD is categorized into nonalcoholic fatty liver (NAFL) and nonalcoholic steatohepatitis (NASH). NAFL is a mostly benign, nonprogressive clinical entity, while NASH can progress to cirrhosis or even hepatocellular carcinoma (HCC)
1. Fat deposition in the liver is histologically significant at 5% or more
2. NASH is histologically characterized by hepatic steatosis associated with evidence of liver cell injury (ballooning degeneration) and inflammation
3. NAFL and NASH are not completely different diseases. Some NAFL patients show slow progression of hepatic fibrosis
4. The upper limit of alcohol drinking is 30 g/day in males and 20 g/day in females
5. Hepatic steatosis induced by drugs is treated as a drug-induced liver disorder
6. Reye’s syndrome and acute fatty liver of pregnancy, which show microvesicular steatosis, are excluded from NAFLD
7. In NASH cirrhosis, certain histological characteristics of NASH, such as hepatic steatosis and ballooning degeneration, are sometimes lost and this is known as “burned-out NASH.”

*The most important factor in the prognosis is hepatic fibrosis, and follow-up and treatment methods should be selected depending on the degree of hepatic fibrosis

NAFLD is characterized by evidence of hepatic steatosis as determined by either imaging or histology, associated with any metabolic factor. Other liver diseases, such as alcoholic liver disease, viral liver disease, and drug-induced liver disorder, are excluded.

The European Association for the Study of the Liver (EASL) defines NAFLD as being characterized by “excessive hepatic fat accumulation associated with insulin resistance (IR)” [5]. In Japan, 60–70% of NAFLD cases are complicated with diabetes mellitus or IR, while others may be complicated with obesity, hypertension, or dyslipidemia without IR [6, 7]. Therefore, we defined NAFLD as “hepatic steatosis associated with any metabolic factor.” Another issue is NAFL progression. McPherson et al. [8] report that of 27 NAFL patients, 12 (44%) had progressed to NASH at the second biopsy. NAFL and NASH are not completely different diseases; there is a certain amount of overlap. Therefore, we specifically state in the guidelines that “NAFL and NASH are not completely different diseases. Some NAFL patients show slow progression of hepatic fibrosis.” In addition, the American Association for the Study of Liver Diseases (AASLD) [9] and EASL management guidelines [5] state that hepatic steatosis induced by drugs should be excluded from NAFLD. We, therefore, specify that “hepatic steatosis induced by drugs is treated as a drug-induced liver disorder, not as NAFLD.”

After the publication of the 2014 guidelines, many cohort studies demonstrated that hepatic fibrosis, but no other histological features of NAFLD, was associated independently with long-term overall mortality, liver transplantation, and liver-related events in NAFLD patients [10–12]. Therefore, we added the following statement to the revised guidelines: “the most important factor in the prognosis is hepatic fibrosis, and follow-up and treatment methods should be selected depending on the degree of hepatic fibrosis.”

We also added new clinical questions (CQs).

## CQ. Which histological factor is most important in assessing survival?

- The stage of hepatic fibrosis is strongly associated with both total and liver-related mortalities. It is important to evaluate the grade of hepatic fibrosis in NAFLD patients. (Evidence Level A, Strength 1).

## Flowchart for the follow-up and screening of hepatic fibrosis, HCC, and cardiovascular disease

**Fig. 1.**
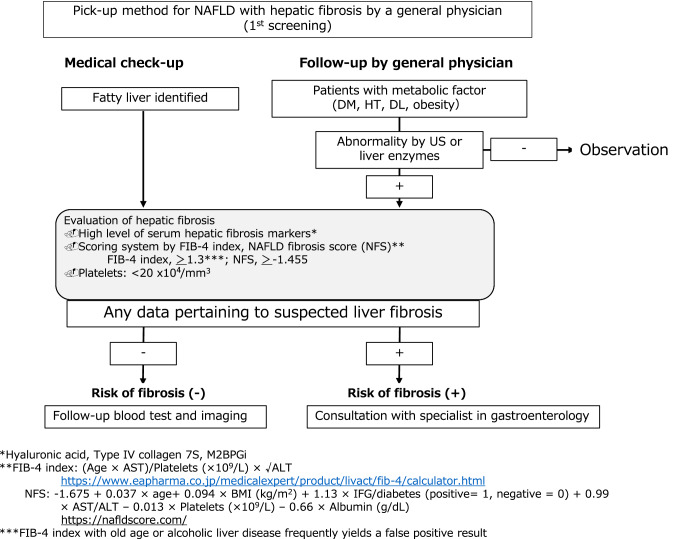
1st Screening system for NAFLD with hepatic fibrosis: flowchart. First screening system for NAFLD is performed by a general physician or medical checkup. If any data indicate liver fibrosis, consultation with a gastroenterology specialist should be considered. *FIB-4 index* Fibrosis-4 index; *NFS* NAFLD fibrosis score; *DM* diabetes mellitus; *HT* hypertension, *DL* dyslipidemia; *M2BPGi* Mac-2 Binding Protein Glycosylation isomer; *BMI* body mass index; *IFG* impaired fasting glucose; *US* ultrasonography

There is currently no overall flowchart for the screening and follow-up of hepatic fibrosis, HCC, or cardiovascular disease (CVD). As noted, the most important factor in the prognosis is hepatic fibrosis. We, therefore, propose that the first step on the flowchart be a screening system for NAFLD by a general physician (Fig. 1). Castera et al. [13] report that simple inexpensive and widely available serum biomarkers, such as the FIB-4 index or the NAFLD fibrosis score (NFS), which have a high negative predictive value for ruling out advanced fibrosis, should be used as the first line. Patients with low risk of advanced fibrosis (FIB-4 < 1.3 or NFS < − 1.455) do not need further assessment, while liver stiffness should be measured by vibration-controlled transient elastography (VCTE) in those with intermediate risk (FIB-4 = 1.3–3.25 or NFS = − 1.455–0.672) or high risk (FIB-4 > 3.25 or NFS > 0.672).

Following Castera et al. [13], we propose a screening method for NAFLD with hepatic fibrosis by a general physician (Fig. 1). At the first screening for NAFLD, physicians are recommended to measure serum hepatic fibrosis markers, such as hyaluronic acid, Type IV collagen 7S, Mac-binding protein glycosylation isomer, and to use a fibrosis scoring system such as the FIB-4 index, NFS, or platelet count. If any data indicate liver fibrosis, consultation with a gastroenterology specialist should be considered.

**Fig. 2.**
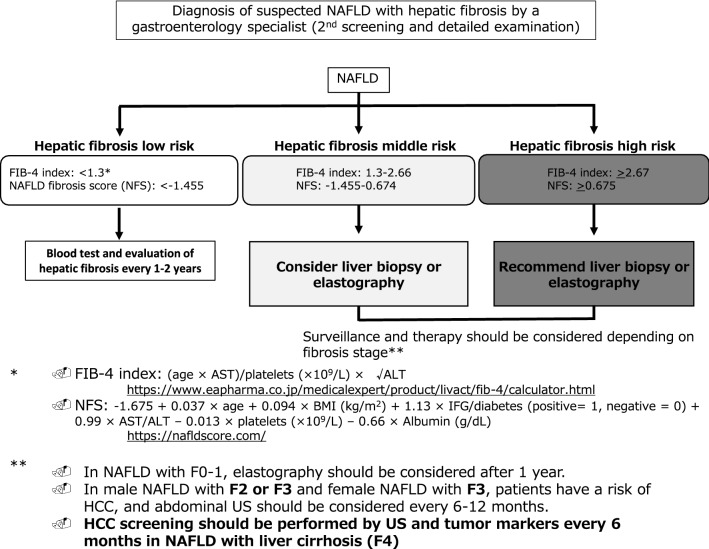
2nd screening system for NAFLD with hepatic fibrosis and HCC screening: flowchart. Second screening is conducted by a gastroenterology specialist. First, the FIB-4 index or NFS is calculated. If the FIB-4 index is increased, liver biopsy or vibration-controlled transient elastography (VCTE) or magnetic resonance elastography (MRE) should be considered or recommended. *FIB-4 index* Fibrosis-4 index; *NFS* NAFLD fibrosis score; *DM* diabetes mellitus; *BMI* body mass index; *IFG* impaired fasting glucose; *US* ultrasonography; *HCC* hepatocellular carcinoma

Figure 2 is a flowchart of the next screening, to be conducted by a gastroenterology specialist. First, the FIB-4 index or NFS is calculated. If the FIB-4 index is under 1.3 or the NFS is under − 1.455, the patient is at low risk of having fibrosis and should repeat the evaluation every 1 year. If moderate hepatic fibrosis (FIB-4 index of 1.3–2.66 or NFS of − 1.455–0.674) is suspected, liver biopsy or VCTE or magnetic resonance elastography (MRE) should be considered. If severe hepatic fibrosis (FIB-4 index: ≥ 2.67 or NFS: ≥ 0.675) is suspected, liver biopsy or elastography is recommended and, depending on fibrosis stage or liver stiffness, surveillance and therapy may be considered. The most important point is to evaluate hepatic fibrosis stage by liver biopsy, elastography, or some other method.

Regarding HCC surveillance, we recommend that in NAFLD with F0-1, the evaluation for fibrosis stage should be performed after 1 year without HCC screening. In male NAFLD with F2 or F3 and female NAFLD with F3, patients have a mild risk of HCC and abdominal ultrasound (US) should be considered every 6–12 months.

HCC screening should be performed by US and tumor markers every 6 months in NAFLD with liver cirrhosis (LC; F4). However, the best screening method in terms of cost and manpower remains to be determined. Loomba et al. [14] recently reported on the American Gastroenterological Association (AGA) Clinical Practice Update on screening and surveillance for HCC in patients with NAFLD. Screening for HCC should be considered in patients with LC or advanced fibrosis. In the revised guidelines, we have added new CQs regarding HCC follow-up and screening in NAFLD/NASH patients.

## CQ. How should NAFLD/NASH patients be followed up?

- Depending on the grade of hepatic fibrosis, it is important to follow-up not only liver-related events, but also CVD and extrahepatic malignancies. (Evidence Level A, Strength 1).

## CQ. How should NAFLD/NASH patients be screened for HCC?

- HCC screening should be performed depending on the stage of hepatic fibrosis and risk factors for HCC. However, the best screening method in terms of cost and manpower remains to be determined. (Evidence Level C, Strength 2).

In terms of HCC risk factors, old age, male, advanced fibrosis, diabetes, and moderate alcohol intake have been reported to be risk factors for NAFLD-HCC [15–17].

CVD, the most common death etiology in NAFLD patients, is another issue. Francque et al. [18] reported the screening method for CVD in NAFLD patients. It is important to note that NASH and NAFLD with hepatic fibrosis increase the risk of cardiovascular event [19, 20]. In cases with hepatic fibrosis, CVD screening is recommended.

**Fig. 3:**
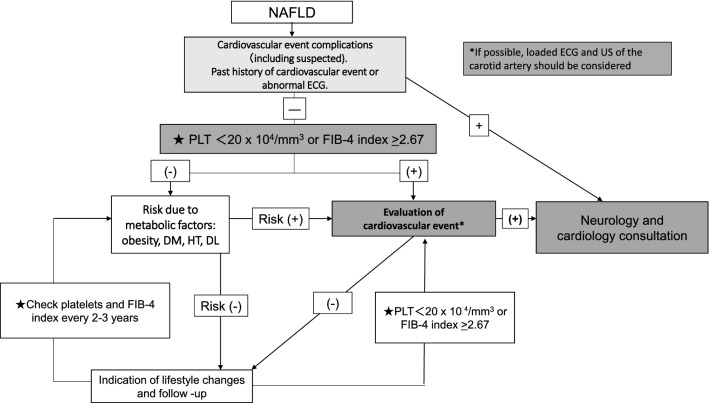
Flowchart for cardiovascular event screening in NAFLD patients. We have to check for cardiovascular disease (CVD) complications and/or a past history of CVD, and perform an electrocardiogram (ECG). If any abnormality is found, we consult a specialist in cardiology or neurology. In NAFLD with a reduced platelet count or increased FIB-4 index, we should evaluate risk based on cardiovascular examination, such as loaded ECG and/or US of the carotid artery. *FIB-4 index* Fibrosis-4 index; *PLT* platelet, *DM* diabetes mellitus; *HT* hypertension, *DL* dyslipidemia; *US* ultrasonography; *ECG* electrocardiogram

Therefore, we propose the flowchart for cardiovascular event screening in NAFLD patients shown in Fig. 3. We first check for CVD complications and/or a past history of CVD, and perform an electrocardiogram (ECG). If any abnormality is found, we then consult a specialist in cardiology or neurology. In NAFLD with a reduced platelet count or increased FIB-4 index, we should evaluate risk based on cardiovascular examination, such as loaded ECG and/or US of the carotid artery. CQ5-3 concerns cardiovascular events in NAFLD patients.

## CQ. Do NAFLD/NASH patients have an increased rate of cardiovascular events?

- In NAFLD/NASH, cardiovascular events are increased, especially in the advanced hepatic fibrosis group. Examination for and evaluation of CVD should be considered in NAFLD patients with advanced fibrosis. (Evidence Level A, Strength 1).

## Epidemiology

The reported prevalence of NAFLD [6, 21–23] varies widely depending on the population studied and the definition or method used. Age, gender, and ethnic differences have an effect on both the prevalence and severity of NAFLD/NASH [6, 21–24]. These differences are also seen in the prevalence of obesity and metabolic syndrome. The prevalence of NAFLD is reported to be 20–40% in Western countries and 12–30% in Asian countries [1, 25]. Annual health checkup data show that 9–30% of Japanese adults have US-diagnosed NAFLD, and NASH is diagnosed in 10–20% or more of NAFLD cases [6, 25, 26]. The estimated prevalence of NASH is approximately 3–5% worldwide [27, 28]. NAFLD/NASH is predominantly diagnosed in middle-aged men and postmenopausal women [28]. A Markov model has estimated there to be approximately 22,600,000 NAFLD patients in Japan and 3,700,000 NASH patients, and by 2030, the prevalence of NASH and advanced fibrosis will have increased [3].

Regarding the rate of HCC development from NAFLD, Younossi et al. [1] report global data showing that the incidence of HCC among NAFLD and NASH patients is 0.44 per 1000 person-years (range 0.29–0.66) and 5.29 per 1000 person-years (range 0.75–37.56), respectively. It is higher in LC due to NAFLD, at 2–3% per year [16, 17].

## Pathogenesis and genomic background

Given the important role of insulin resistance and oxidative stress in the pathophysiology of NAFLD/NASH [29–31], several studies have attempted to investigate their effects.

The I148M variant of patatin-like phospholipase domain-containing 3 (*PNPLA3*) is widely known to be associated with the occurrence and progression of NAFLD/NASH worldwide [32–34]. The mechanism remains unknown, however, it has been suggested that the 148M variant disrupts ubiquitylation and proteasomal degradation of *PNPLA3*, resulting in an accumulation of PNPLA3-148M and impaired mobilization of triglycerides (TGs) from lipid droplets (LDs) [35–37]. There is known to be a significant association between HCC and the 148M variant [38, 39]. *TM6SF2, GCKR, GATAD2A* and *DYSF* have been reported as genomic background candidates [39–43]. In addition, there has been a recent focus on the relationship between NAFLD and gut dysbiosis [44, 45]. Henao-Mejia et al. [46] found that inflammasome-mediated dysbiosis regulates the progression of NAFLD and obesity, however, a more detailed analysis using human samples is needed. Sarcopenia has also been examined [47].

## Diagnosis and imaging

The diagnosis of NAFLD is described as part of the concept and definition of NAFLD (Table 1). ‘‘Nonalcoholic’’ is defined as an upper limit of alcohol drinking of 30 g/day in males and 20 g/day in females. NASH is diagnosed from liver biopsy on the basis of the presence of steatohepatitis, however, liver biopsy has several drawbacks. It is an expensive and invasive procedure and there is potential for sampling error and variability in interpretation by pathologists [48, 49]. However, liver biopsies remain the gold standard for the diagnosis of NASH and are, therefore, often recommended, particularly in NAFLD with suspected advanced fibrosis and in suspected coexisting chronic liver disease, where there is a need to distinguish NASH from other chronic liver diseases [9].

Regarding the noninvasive assessment of NASH and advanced fibrosis, there are no practically useful surrogate markers for diagnosing NASH. Platelet count [50] and scoring systems such as the NFS [51] and the FIB-4 index [52, 53] have proven to be useful for predicting fibrosis in the Japanese population and worldwide. However, care must be taken, because age is one of the factors in the FIB-4 index, and higher FIB-4 index titers in older patients do not necessarily reflect actual liver stiffness or fibrosis grade [54, 55].

A number of imaging modalities can detect liver fat and liver stiffness [56–58], including VCTE, which measures liver stiffness noninvasively, has shown promising results for assessing the severity of liver fibrosis. Recently, MRE was found to have statistically significantly higher diagnostic accuracy than VCTE in the detection of each stage of fibrosis [59]. MRE and VCTE each have a role to play in the detection of fibrosis in patients with NAFLD, depending on the level of accuracy desired [60].

## Therapy

**Fig. 4:**
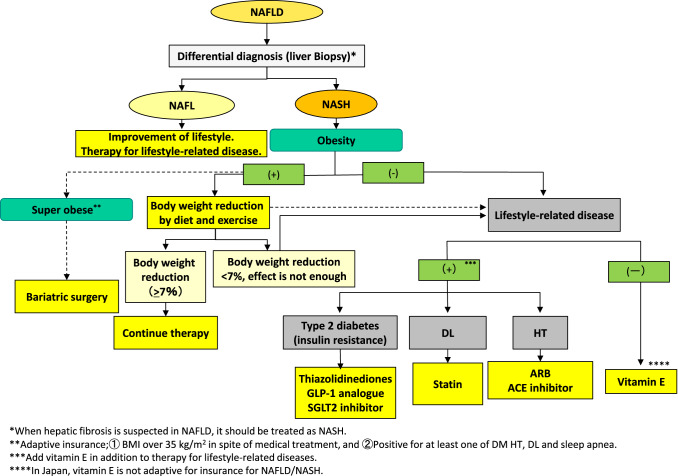
Flowchart of therapy for NAFLD/NASH. *BMI* body mass index; *DM* diabetes mellitus; *HT* hypertension, *DL* dyslipidemia; *GLP-1* glucagon-like peptide-1; *SGLT2* sodium glucose co-transporter; *ARB* angiotensin II receptor antagonist; *ACE* angiotensin II converting enzyme inhibitor

Figure 4 is a flowchart of therapy for NAFLD patients. It is similar to that in the previous guidelines [4]. NAFLD is usually associated with metabolic disturbance such as visceral obesity, insulin resistance, type 2 diabetes mellitus, or dyslipidemia, and these underlying conditions play a crucial role in its pathogenesis. Therefore, it makes sense to treat not only the liver disease itself but also these associated metabolic morbidities, and it is likewise essential to prevent stimulation or pathogenesis of these diseases, so-called “2nd hits,” in the management of NAFLD/NASH [61]. Treatments for these associated conditions include lifestyle modification, weight loss, and increased physical activity, all of which have been shown to be effective and represent the cornerstone of treatment [61].

Lifestyle-related interventions such as diet and exercise therapies have been reported to improve serum transaminase levels as well as liver fat as measured by US and magnetic resonance imaging (MRI) in NAFLD patients [62, 63]. Musso et al. [64] evaluated the effects of weight loss in NAFLD in eight randomized controlled trials, four of which included posttreatment histology. Their meta-analysis showed that a 5% or greater weight loss improved hepatic steatosis, and a 7% or greater weight loss correlated with improvement in the NAFLD Activity Score (NAS). Unfortunately, only 50% of subjects were able to attain a weight loss of 7% or greater even with significant intervention. Vilar-Gomez et al. [65] evaluated the effects of weight loss through lifestyle modifications in 261 patients with paired liver biopsies, finding that the degree of weight loss was associated independently with improvements in all NASH-related histology. Furthermore, for those individuals who lost 10% or more of their body weight, 45% experienced regression of fibrosis, 90% had resolution of steatohepatitis, and 100% demonstrated improvements in NAS. These findings indicate the effectiveness of body weight reduction for patients with obesity-related NAFLD/NASH. In almost all reports of dietary interventions for patients with obesity-related NAFLD, a low-calorie diet is prescribed and, in terms of dietary contents, the proportions of energy intake from carbohydrates and lipids are often restricted to 50–60% and 20–25%, respectively. Recent attention has focused on low-carbohydrate and Mediterranean diets [66, 67], and coffee intake has been reported to inhibit hepatic fibrosis and hepatocarcinogenesis [68, 69].

Exercise therapy is another useful lifestyle-related intervention for NAFLD/NASH. Consistent aerobic exercise in 30- to 60-min sessions held 3–4 times weekly for 4–12 weeks in patients with NAFLD complicated by obesity has been shown to improve liver fat content, even without accompanying body weight reduction [70, 71]. Oh et al. [72] report that physical activity of moderate to vigorous intensity for ≥ 250 min/week as part of lifestyle management improves NAFLD pathophysiology in obese men in Japan. The benefits seem to be acquired through reducing inflammation and oxidative stress levels and altering the fatty acid metabolism. While these reports do not examine histological changes, liver fat are thought to improve even with exercise therapy alone.

## CQ. What kind of diet is recommended to improve NAFLD/NASH?

- Body weight reduction through a low-calorie diet improves liver function and fatty changes in the liver in patients with NAFLD. To improve NAFLD/NASH, we recommend prioritizing the optimization of energy intake and restricting lipids or carbohydrates in terms of the proportions of nutrient intake. (Evidence Level C, Strength 2).

## CQ. Is exercise beneficial for improving NAFLD/NASH?

- Although the effects of exercise on liver histology have not been fully clarified, we recommend implementing exercise therapy because, even alone, it improves liver function and liver fat in the liver in patients with NAFLD. (Evidence Level B, Strength 2).

## Pharmacological treatments

Vitamin E and pioglitazone have been shown to improve liver function and liver histological findings [73–76]. However, their safety over the long term remains to be evaluated.

## CQ. Is vitamin E effective for patients with NAFLD/NASH?

- Vitamin E improves hepatic biological and histological parameters in patients with NASH, and is recommended. However, its safety over the long term in patients with CVD, congestive heart failure, or bladder cancer has not yet been fully assessed.

## CQ. Are thiazolidinediones effective for patients with NAFLD/NASH?

- Pioglitazone is recommended for NASH patients with insulin resistance. (Evidence Level A, Strength 2).

Other studies have focused on other diabetic drugs such as incretin-related drugs like glucagon-like peptide-1 (GLP-1) receptor analogue [77–80] dipeptidyl peptidase 4 (DPP-4) inhibitor [81, 82], and sodium glucose co-transporter 2 (SGLT2) inhibitor [83–85]. Both GLP-1 analogue and SGLT2 inhibitor not only decrease body weight but also improve the glucose metabolism. As SGLT2 inhibitor is known to be effective to heart failure [86], additional effects can reasonably be expected in NASH patients. Some reports have demonstrated that GLP-1 analogue and SGLT2 improve liver function and liver histological findings [79, 80, 85, 87, 88]. In the revised guidelines, two drugs have been added.

## CQ. Is SGLT2 inhibitor effective for NAFLD/NASH?

- In NAFLD/NASH patients with type 2 diabetes, SGLT2 inhibitor improves liver enzymes and histological findings, and its administration is, therefore, suggested. SGLT2 inhibitor is not adaptive for insurance of NAFLD/NASH. (Evidence Level C, Strength 2).

## CQ. Are incretin-related drugs such as GLP-1 analogue and/or DPP-4 inhibitor effective for NAFLD/NASH?

- In NAFLD with type 2 diabetes, GLP-1 analogue has been found to improve liver function and liver histological findings. SGLT2 inhibitor is not adaptive for insurance of NAFLD/NASH. The effect of DPP-4 inhibitor is not constant. (Evidence Level C, Strength 2).

Regarding to both drugs, randomized trials with greater numbers of patients are needed.

Drugs for dyslipidemia and hypertension are reported to improve liver enzymes [89–93], as described in previous guideline.

## CQ. Are drugs for dyslipidemia effective for patients with NAFLD/NASH?

- HMG-CoA reductase inhibitors (statins) are recommended for NAFLD/NASH patients with hypercholesterolemia. However, the effect of ezetimibe is not constant. (Evidence Level C, Strength 2).

## CQ. Is an angiotensin II receptor antagonist (ARB) or angiotensin II converting enzyme inhibitor (ACE) effective for hypertensive patients with NAFLD/NASH?

- We recommend an ARB or ACE for NASH patients with hypertension. (Evidence Level C, Strength 2).

Ursodeoxycholic acid (UDCA) and biguanides have no significant effect on liver histology and we do not recommend them as a specific treatment for liver disease in patients with NASH [94–96], as described in previous guideline.

## BQ. Are conventional doses of ursodeoxycholic acid effective for patients with NAFLD/NASH?

- We do not recommend UDCA at conventional dose levels for the treatment of NAFLD/NASH.

## CQ. Is biguanides effective for patients with NAFLD/NASH?

- We do not recommend biguanides for the treatment of NAFLD/NASH, because there is no evidence, suggesting improvement of liver enzyme and liver histology in NAFLD/NASH patients. (Evidence Level B, Strength 2).

## Other candidate drugs

### FRQ. Are there any other effective drugs for treatment of patients with NAFLD/NASH in the future?

There are several ongoing drug trials for NAFLD/NASH therapy, including trials of obeticholic acid (OCA), elafibranor, selonsertib (SEL), apoptosis signal-regulating kinase 1 (ASK1), cenicriviroc (CVC), fibroblast growth factor (FGF)-21, Aramchol, acetyl-CoA carboxylase (ACC) inhibitor (GS-0976), FGF19 (NGM-282), pemafibrate, emricasan, toll-like receptor 4 (TLR4) inhibitor (JKB-21), solithromycin, SSAO/VAP-1 inhibitor (BI 1467335), IMM-124E, galectin-3 inhibitor (GR-MD-02), and heat shock protein (HSP) 47 [97–108] to be addressed in future research questions.

Nevertheless, the trials for NAFLD/NASH had several problems. First, even in placebo groups, about 20% of patients improved due to lifestyle improvements. Second, to confirm improvement of liver fibrosis, which associated with prognosis, an observation period of 5 years or more is necessary. In addition, histological evaluation differs among pathologists. It is also problematic that NAFLD is a syndrome, and therefore, includes many pathogeneses. The effects of drugs are, therefore, not always constant. Finally, the prevalence of the *PNPLA3* variant, which is a risk factor of progression of NASH, is different among races. It is expected that these problems will be resolved, and that new drugs will be approved for NASH therapy.

Elucidating the pathogenesis of NAFLD/NASH and developing therapies are now worldwide issues, and it is important that Japanese medical studies of NAFLD/NASH advance. For that purpose, these guidelines were prepared by searching for relevant evidence worldwide without regard to ethnic characteristics, and the obtained evidence was then summarized from a Japanese perspective. Of course, ethnic differences correlate with susceptibility to metabolic syndrome-related diseases including NAFLD/NASH [109], possibly as a result of genomic polymorphism. Therefore, it is also important to develop Japanese-based clinical research work and to interpret other countries’ evidence in light of ethnic considerations. The revised guidelines not only summarize the current clinical state of NAFLD/NASH but also point to future directions for study.

## Appendix

The members of the Guidelines Committee who created and evaluated the Japanese Society of Gastroenterology “Evidence-based clinical practice guidelines for nonalcoholic fatty liver disease/nonalcoholic steatohepatitis2020” are listed below.

## Creation Committee

Chair: Katsutoshi Tokushige (Institute of Gastroenterology, Department of Internal Medicine, Tokyo Women’s Medical University). Vice-Chair: Kenichi Ikejima (Department of Gastroenterology, Juntendo University Graduate School of Medicine). Members: Masafumi Ono (Division of Gastroenterology and Hepatology, Department of Internal Medicine, Tokyo Women’s Medical University Medical Center East), Yuichiro Eguchi, (Loco Medical General Institute), Yoshihiro Kamada, (Department of Gastroenterology and Hepatology, Osaka University Graduate School of Medicine), Yoshito Itoh (Department of Gastroenterology and Hepatology, Graduate School of Medicine, Kyoto Prefectural University of Medicine), Norio Akuta (Department of Hepatology, Toranomon Hospital), Masato Yoneda (Department of Gastroenterology and Hepatology, Yokohama City University Graduate School of Medicine), Motoh Iwasa (Department of Gastroenterology and Hepatology, Mie University Hospital), Masashi Yoneda (Division of Hepatology and Pancreatology, Department of Internal Medicine, Aichi Medical University), Motoyuki Otsuka (Department of Gastroenterology, Graduate School of Medicine, The University of Tokyo), Nobuharu Tamaki (Department of Gastroenterology and Hepatology, Musashino Red Cross Hospital), Tomomi Kogiso (Institute of Gastroenterology, Department of Internal Medicine, Tokyo Women’s Medical University).

## Evaluation Committee

Chair: Yoshiyuki Takei (Department of Gastroenterology and Hepatology, Mie University Graduate School of Medicine). Vice-Chair: Hitoshi Yoshiji (Third Department of Internal Medicine, Nara Medical University). Members: Masataka Seike (Department of Gastroenterology, Oita Cardiovascular Hospital), Sumiko Nagoshi (Department of Gastroenterology and Hepatology, Saitama Medical Center, Saitama Medical University).

## The Japanese Society of Gastroenterology

President: Kazuhiko Koike (Kanto Central Hospital).

Past President: Tooru Shimosegawa (South Miyagi Medical Center).

Director Responsible: Hiroto Miwa (Division of Gastroenterology, Department of Internal Medicine, Hyogo College of Medicine), Nobuyuki Enomoto (First Department of Internal Medicine, Faculty of Medicine, University of Yamanashi).

## The Japan Society of Hepatology

President: Tetsuo Takehara (Department of Gastroenterology and Hepatology, Osaka University Graduate School of Medicine).

Past President: Kazuhiko Koike (Kanto Central Hospital).

Director Responsible: Kazuaki Chayama (Department of Gastroenterology and Metabolism, Graduate School of Biomedical Sciences, Hiroshima University).
